# P-1759. Antibiotic De-Escalation in Neutropenic Fever

**DOI:** 10.1093/ofid/ofae631.1922

**Published:** 2025-01-29

**Authors:** Michelle Evans, Douglas Tremblay, Meenakshi M Rana, Samantha E Jacobs, Risa Fuller

**Affiliations:** Newark Beth Israel Medical Center, Piscataway, New Jersey; Mount Sinai Hospital, New York, New York; Icahn School of Medicine at Mount Sinai, Scarsdale, NY; Icahn School of Medicine at Mount Sinai, Scarsdale, NY; Icahn School of Medicine at Mount Sinai, Scarsdale, NY

## Abstract

**Background:**

Cancer patients with neutropenic fever are frequently continued on broad-spectrum empiric antimicrobial therapy (EAT) until neutrophil recovery, but recent randomized trials demonstrate that EAT de-escalation prior to neutrophil recovery is safe in certain subgroups. We evaluated outcomes of EAT de-escalation based on clinical criteria in patients with neutropenic fever at our institution.

**Methods:**

We retrospectively reviewed charts of 49 patients with acute myeloid leukemia (AML) or hematologic stem cell transplantation (HSCT) and neutropenic fever following revisions to Standard of Practice (SOP) institutional guidelines (hereafter “SOP” group) allowing for antibiotic de-escalation using a clinical approach in comparison to a historic cohort of 60 patients. The revised SOP recommended antibiotic de-escalation to levofloxacin prophylaxis in patients who received *≥* 72 hours of EAT, were afebrile and clinically stable for 48 hours, and did not have a clinically or microbiologically documented infection. The primary endpoint was the EAT duration. Secondary end points included incidence of recurrent fever, infections, vasopressor support and/or intensive care unit (ICU) stay, and in-hospital mortality.

**Results:**

Baseline characteristics were similar between both groups, with differences including a larger proportion of males in the SOP group compared to the control group (59% vs. 35%) and the primary underlying malignancy of multiple myeloma in the SOP group compared to AML in the control group. The median duration of EAT prior to de-escalation was shorter in the SOP group compared to the historic cohort (5.0 [IQR 4.0, 6.0] vs. 7.0 days [IQR 5.0, 10.2], p< 0001). There was no difference in incidence of recurrent fever (20% vs. 30%, p=0.3) or secondary infection (10% vs. 12%, p=0.8). Incidence of vasopressor support and/or ICU stay(10% vs. 3.3%, p=0.2) and in-hospital mortality (2.0% vs. 3.3 %, p >0.9) were also similar between both groups.

**Conclusion:**

Our study indicates that our new institutional guidelines successfully lead to reduced broad spectrum antimicrobial exposure without increased infections, ICU stays, or mortality. Thus, de-escalation of EAT based on clinical criteria is safe in this high risk patient population.

**Disclosures:**

**Douglas Tremblay, MD**, AbbVie: Advisor/Consultant|Astellas: Grant/Research Support|Cogent Biosciences: Advisor/Consultant|Cogent Biosciences: Grant/Research Support|Gilead: Grant/Research Support|GSK: Advisor/Consultant|Novartis: Advisor/Consultant|Sierra Oncology: Advisor/Consultant|Sobi: Advisor/Consultant|Sobi: Grant/Research Support|Sobi: Patent for use of pacritinib and azacitidine in CMML|Sumitomo: Grant/Research Support **Samantha E. Jacobs, MD, MS**, Ansun Biopharma: Advisor/Consultant|Eurofins, Viracor, LLC.: Grant/Research Support

